# Audit of Assessment and Documentation of Sexual Dysfunction in Liaison Psychiatry Service

**DOI:** 10.1192/bjo.2026.11829

**Published:** 2026-06-30

**Authors:** Muhammad Abdur Rehman, Doorgadharshanee Ramdin Doorga, Rony Mathews, Oluwatobiloba Alamu, Fareedoon Ahmed

**Affiliations:** 1 Kent and Medway Mental Health NHS Trust, Kent, United Kingdom; 2 Dartford and Gravesham NHS Trust, Kent, United Kingdom

## Abstract

**Aims:**

Sexual dysfunction is a commonly encountered adverse effect associated with the treatment of psychotropic drugs, particularly antidepressants and antipsychotics. This adverse effect causes significant challenges, as it can severely impact the quality of life and treatment compliance among patients.

This audit aims to evaluate the extent to which sexual dysfunction is assessed, discussed, and documented for adult patients assessed by a Liaison Psychiatry service and commenced on psychotropic medication.

**Methods:**

A retrospective clinical audit was conducted of consecutive patient assessments undertaken by the Liaison Psychiatry service over six months from March 2025 to August 2025.The total number of patients was 1214, out of which 97 were started on psychotropic medication.

Inclusion criteria were. 1. patients ≥18 years of age 2. those who were assessed and commenced on, or reviewed for initiation of, psychotropic medication.

Exclusion criteria were those who were not started on psychotropic medication or were already established on psychotropics before assessment.

Clinical records were reviewed to identify documentation of discussion regarding sexual dysfunction, potential medication-related sexual side effects, and evidence of informed consent. All data were fully anonymised, and no identifiable patient information was recorded.

The audit standard was set at 100%, requiring documentation of the presence or absence of sexual dysfunction and/or evidence that advice regarding potential sexual side effects was provided at the time of medication initiation, in accordance with NICE guidance and local trust Policies.

**Results:**

The audit findings revealed

- Consent for psychotropic medication initiation was documented in only 64% of patients.
- Discussion of sexual side effects of medication was documented in only 4% of patients
- Discussion and documentation of other side effects of medication were completed in only 19% of patients

**Conclusion:**

These findings demonstrated significant gaps in assessment and documentation practice within the Liaison Psychiatry service and a lack of compliance with NICE guidelines. Addressing these deficits within the Liaison Service through clear documentation and education is vital to ensure patient-centred care in line with national guidelines and local trust policies.

A re-audit will be conducted within 8 months after recommendations are put in place, to assess improvement in compliance withNICE guidelines and local Trust Policies.

